# Exploring Ternary Essential Oil Mixtures of Moroccan Artemisia Species for Larvicidal Effectiveness Against *Culex pipiens* Mosquitoes: A Mixture Design Approach

**DOI:** 10.1155/japr/2379638

**Published:** 2025-02-20

**Authors:** Abdellatif Alami, Yassine Ez zoubi, Mouhcine Fadil, Saoussan Annemer, Mohammed Bassouya, Wafae Moustaid, Abdellah Farah

**Affiliations:** ^1^Laboratory of Applied Organic Chemistry, Faculty of Sciences and Techniques, Sidi Mohamed Ben Abdellah University, Fez, Morocco; ^2^Laboratory of Research and Development in Engineering Sciences, Biotechnology Environmental Technology and Valorization of Bio-Resources Team, Department of Biology, Abdelmalek Essaadi University, Tetouan, Morocco; ^3^Laboratory of Biotechnology, Environment Agrifood and Health, Faculty of Sciences Dhar El Mahraz, Sidi Mohamed Ben Abdellah University, Fez, Morocco

**Keywords:** artemisia, bioinsecticides, *Culex pipiens*, essential oils, mixture design

## Abstract

Essential oils (EOs) have gained significant attention for their biopesticidal properties in pest management. This study investigates the insecticidal potential of EOs extracted from the aerial parts of three indigenous *Artemisia* species—*Artemisia absinthium*, *Artemisia arborescens*, and *Artemisia campestris*—collected from various provenances in Morocco. The EOs were tested individually and in combination against *Culex pipiens* (*C. pipiens*) larvae to explore potential synergistic interactions using a mixture design methodology. Gas chromatography–mass spectrometry (GC-MS) and gas chromatography with flame ionization detection (GC-FID) analyses revealed quantitative and qualitative variations in the chemical composition of the oils. The primary constituents of *A. absinthium* were identified as thujone (32.20% ± 2.65%), camphor (19.95% ± 2.64%), and chamazulene (19.58% ± 2.33%). In *A. arborescens*, thujone (52.05% ± 3.84%), camphor (10.71% ± 3.08%), and eucalyptol (4.79% ± 1.53%) were the major components, while *A. campestris* comprised camphor (18.98% ± 2.65%), car-3-en-5-one (11.25% ± 2.33%), and thujone (6.63% ± 1.67%). When applied individually, all three EOs exhibited significant larvicidal activity against *C. pipiens* larvae, with *A. arborescens* showing the highest efficacy (LC_50_ 11.11 *μ*g/mL (5.45 ± 22.62)) compared to *A. absinthium* (LC_50_: 16.98 *μ*g/mL (6.73 ± 27.39)) and *A. campestris* (LC_50_: 19.07 *μ*g/mL (13.57 ± 23.38)). In mixture experiments, the mixture design outcomes reveal that the ternary formulation comprising 58% *A. absinthium*, 26% *A. arborescens*, and 16% *A. campestris* emerged as the most effective blend, achieving complete larval eradication. This study highlights the potential of *Artemisia* EO combinations as a sustainable and effective alternative for managing mosquito vectors of disease.

## 1. Introduction

Mosquitoes, often dismissed as mere nuisances, play a critical role as vectors of numerous diseases [1]. These small insects are among the deadliest creatures on Earth, transmitting dangerous pathogens to humans and animals [2, 3]. Female mosquitoes are primarily responsible for disease transmission, as they require blood meals for reproduction. During this process, they acquire pathogens from infected hosts and pass them on to others during subsequent feedings. Prominent mosquito genera, such as *Aedes*, *Anopheles*, and *Culex*, are vectors for a range of severe infections, including chikungunya virus, Japanese encephalitis, filariasis, malaria, yellow fever, and West Nile virus [4, 5]. The symptoms of these diseases range from mild fevers to severe complications and, in some cases, fatal outcomes.

*Culex pipiens* (*C. pipiens*) (the common house mosquito) is one of the most widespread mosquito species globally and has been implicated in disease transmission in various regions [6], including Morocco [7]. It is a known vector of the West Nile virus [8] and causes significant nuisance to humans, leading to allergic reactions such as localized skin irritation and, in severe cases, systemic responses like angioedema and urticaria [9]. Given its global distribution and public health impact, there is an urgent need for effective research and interventions to curb the spread of *C. pipiens* and other disease-transmitting mosquitoes.

To manage mosquito populations, chemical insecticides are commonly employed. However, their extensive use has raised concerns about environmental harm, human health risks, and the emergence of insecticide resistance [10–13]. Insecticides such as pyrethroids, organochlorines, organophosphates, and carbamates are frequently used in aquatic environments to target both adult mosquitoes and their larvae [14]. Unfortunately, these chemicals can harm nontarget organisms, including bees, butterflies, and fish, causing ecological imbalances and biodiversity loss [15, 16].

As a sustainable alternative, botanical insecticides derived from natural products, particularly aromatic plants and their essential oils (EOs), have gained significant attention [17]. Recent research has focused on formulating insecticides that are not only environmentally friendly but also cost-effective, biodegradable, and safe for nontarget organisms [18–23]. EOs, with their complex chemical composition (typically comprising 20–60 components) [24], have demonstrated notable mosquito-killing properties. Examples include *Illicium verum* (effective against *Aedes aegypti*), *Myristica fragrans* (effective against *Aedes aegypti*), and *Zingiber officinale* (effective against *Culex quinquefasciatus*) [25–27]. Earlier studies have evaluated the efficacy of plant-derived EOs against *C. pipiens* larvae [28–32], highlighting their potential as natural mosquito control agents due to their low toxicity, affordability, and availability [33–35]. Among the plant genera investigated, *Artemisia* (Asteraceae family) stands out, with over 800 species valued for their medicinal and EO-rich properties [36]. Several studies have assessed the insecticidal potential of *Artemisia* EOs. For instance, kaoutar Abdelali et al. demonstrated the larvicidal and pupicidal effects of *Artemisia herba*-*alba* EO against *C. pipiens* [37]. Similarly, Moroccan studies have shown the effectiveness of EOs from wild and cultivated *Artemisia campestris* against *C. pipiens* larvae [7]. Research by El-Sabrout, Zoghroban, and Abdelgaleil revealed the insecticidal properties of *A. judaica* EO, alongside other plant species against *C. pipiens* [38], while Zahran, Abou-Taleb, and Abdelgaleil identified *A. monosperma* EO as the most potent insecticidal agent among 16 Egyptian botanical EOs [39]. Synergism, the interaction where combined components produce a greater effect than individually [40, 41], is a practical approach to reducing insecticide input [11, 42]. Studies have demonstrated that certain combinations of EOs or their constituents exhibit enhanced toxicity against mosquitoes and other pests compared to single components [3, 43–45].

This study is aimed at evaluating the larvicidal activity of binary and ternary mixtures of EOs derived from the aerial parts of three *Artemisia* species—*A. absinthium*, *A. arborescens*, and *A. campestris*—using a mixture design methodology. Additionally, it seeks to identify their chemical constituents through GC-MS and GC-FID analysis and develop an optimal EO formulation for controlling *C. pipiens* larvae effectively.

## 2. Material and Methods

### 2.1. Plant Material

The Asteraceae species *A. arborescens*, *A. absinthium*, and *A. campestris* were collected from three different provenances between April and June 2022, with full details provided in Table 1. The identification of these species was confirmed by Professor Badr Satrani, a botanist at Morocco's Forestry Research Center in Rabat (FRC-Rabat). To ensure a high concentration of bioactive compounds, the plants were harvested during the flowering stage. The aerial parts of the plants, including stems, leaves, and flowers, were collected to facilitate a comprehensive extraction of EO. After collection, the samples were transported to the laboratory and air-dried in a ventilated, shaded environment for 10 days to preserve their phytochemical integrity. The GPS coordinates of the sampling locations, listed in Table 1, ensure the reproducibility of this study and provide a solid framework for future research. Additionally, a footnote in Table 1 references the detailed procedures for sample preparation and EO extraction, as outlined in the Material and Methods section.

### 2.2. EOs' Extraction

The aerial parts from each species were divided into small portions and placed in a flask containing 1 L of distilled water, with a total sample weight of 100 g. EOs were then extracted using a Clevenger apparatus [46] for 3 h. The extracted EOs were collected and stored at 4°C for subsequent use.

### 2.3. Chromatography Analysis

Gas chromatography–mass spectrometry (GC-MS) and gas chromatography with flame ionization detection (GC-FID) were employed to analyze the chemical composition of the EOs. The GC-MS analysis was performed on a Trace GC Ultra system equipped with a triple quadrupole detector, a splitless injector, and an RTxi-5 Sil MS apolar capillary column (30 m × 0.25 mm ID × 0.25 *μ*m). The operating conditions included temperature ramps of 2 min at 50°C, 2 min at 160°C, and 2 min at 280°C. The Polaris QMS mass spectrometer interface was maintained at 280°C. Quantitative analysis was carried out using a Hewlett–Packard gas chromatograph (HP 6890) fitted with an HP-5 capillary column (30 m × 0.25 mm, 0.25 *μ*m film thickness) and an FID detector. The oven temperature program involved an initial hold of 5 min, followed by a gradual increase to 250°C at a rate of 4°C/min. Nitrogen gas was used as the carrier at a flow rate of 1.8 mL/min. Prior to injection, the EO samples were diluted 1:50 in methanol, and 1 *μ*L of the diluted sample was injected in split mode (split ratio 1:50, flow rate 72.1 mL/min). Identification of phytochemical components was based on retention indices (RIs) calculated using a homologous series of alkanes (C8–C28) and compared with published literature data [47, 48].

### 2.4. Mosquito Larva Collection

*C. pipiens* larvae were collected from a permanent water body at Oued El-Mehraz (altitude: 423 m; 34°02⁣′13.74⁣′′ N, 4°59⁣′59.279⁣′′ W). The larvae were captured using a rectangular plastic dish and reared under controlled conditions in the entomology unit of the Regional Public Health Laboratory of Fez (LRSPF). The rearing environment was maintained at a water temperature of 24.6° ± 2°C and a relative humidity of 50%–70%. For the bioassay experiments, third-instar larvae were selected. Identification of the larvae was performed using the Moroccan identification key for Culicidae [49] and specialized software for identifying African and Mediterranean mosquitoes [50], based on their morphological characteristics.

#### 2.4.1. Larvicidal Bioassays

##### 2.4.1.1. The Toxicity Assessment of the Individual Oils

The larvicidal tests were conducted in accordance with the guidelines established by the World Health Organization, with slight modifications [51]. Preliminary experiments were carried out to determine an appropriate range of concentrations for each EO. For *A. absinthium*, the tested concentrations in ethanol were 1.25, 4.5, 10, 20, 40, 80, and 100 *μ*g/mL. For *A. arborescens* and *A. campestris*, the tested concentrations were 4.5, 8.75, 15, 20, and 30 *μ*g/mL and 4, 8, 12.5, 20, 25, 30, and 50 *μ*g/mL, respectively. All concentrations were prepared in triplicate. In each experiment, a suspension of 1 mL of the EO in ethanol was added to 99 mL of distilled water containing third-instar larvae. A control test was conducted simultaneously, consisting of 20 larvae in 99 mL of distilled water with 1 mL of ethanol. Each experiment, including the control, was performed in triplicate. Larval mortality rates were assessed 24 h after treatment. If the control group exhibited a mortality rate greater than 5%, the mortality rates for larvae exposed to EOs were corrected using Abbott's formula [52]. In cases where control mortality exceeded 20%, the larvicidal tests were repeated to ensure reliability.

### 2.5. Mixture Design and Statistical Analysis

**Figure 1:**
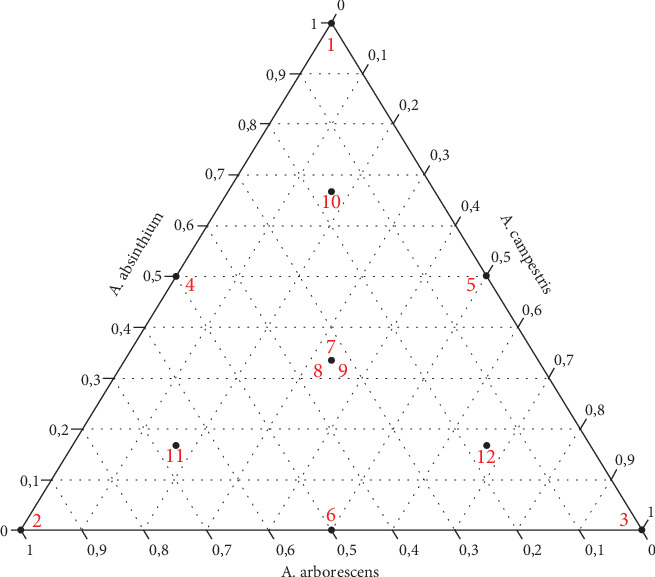
Experimental point positions for the augmented simplex-centroid design.

The mixture design methodology was employed to optimize the insecticidal activity (mortality percentage) against *C. pipiens* larvae while minimizing the number of experiments required. An augmented simplex-centroid design (Figure 1) was selected to identify the optimal formulation by combining three EOs. This matrix comprises the three pure constituents: the half-and-half mixtures, the equiproportional mixture of the three oils, and the mixtures located at the centers of gravity of the unit simplexes [53]. To evaluate the pure error (PE) and compare it with the lack of fit (LOF), Experiment 7 was performed in triplicate. Consequently, the total number of experiments for this design was 12. The proportions of the components in the mixtures adhered to the fundamental constraint of mixture designs, where the sum of all components equals 100%, as illustrated in Equation (1).

(1) $$\begin{array}{c} \overset{n}{\underset{i=1}{\sum }}x_{i}=100\% \end{array}$$

To fit the response in terms of the three components, three models, including linear, quadratic, and special cubic were tested as shown in the following equations.

(2) $$\begin{array}{c} Y_{\text{linear}}=\alpha_{1}X_{1}+\alpha_{2}X_{2}+\alpha_{3}X_{3}+ɛ \end{array}$$

(3) $$\begin{array}{c} Y_{\text{quadratic}}=\alpha_{1}X_{1}+\alpha_{2}X_{2}+\alpha_{3}X_{3}+\alpha_{12}X_{1}X_{2}+\alpha_{13}X_{1}X_{3}+\alpha_{23}X_{2}X_{3}+ɛ \end{array}$$

(4) $$\begin{array}{c} Y_{\text{Special⁣cubique}}=\alpha_{1}X_{1}+\alpha_{2}X_{2}+\alpha_{3}X_{3}+\alpha_{12}X_{1}X_{2}+\alpha_{13}X_{1}X_{3}+\alpha_{23}X_{2}X_{3}+\alpha_{123}X_{1}X_{2}X_{3}+ɛ \end{array}$$

where *Y* is the response variable expressed in % (mortality); *α*_1_, *α*_2_, and *α*_3_ are the coefficients of the linear terms; *α*_12_*α*_23_ and *α*_23_ are the coefficients of the binary terms, *α*_123_, the coefficient of the ternary terms, and, *ɛ*, the error term.

To check the significance of the fitted model, we used the ANOVA test. To do this, we calculated the F_ratio_ mean square regression (MS_R_)/mean square residual (MS_r_), which is the ratio between the mean square due to the regression and the mean square due to the residuals. To test the goodness of fit of the model, the F_ratio_ LOF/PE was calculated, which is the ratio between the mean square due to lack of fit (LOF) and the mean square due to pure error (PE) [54]. The quality of the chosen model was expressed by the value of the coefficient of determination (*R*^2^, adjusted *R*^2^, and predicted *R*^2^). The closer this value is to 1, the better the variability explained by the model than that explained by the residuals [55, 56].

To affirm or reject the significance of factors, the *t*-student test was used at the 95% significance level. In practice, the ratio between the value of the coefficient and the value of its standard deviation is calculated. This ratio is the *t*-student statistic from which the probability of the coefficient being equal to zero can be assessed. In the coefficient table, each factor is associated with *t*-student and *p* value values. The smaller the *p* value, the more statistically significant the coefficient [57].

To complete the optimization step, the contour plot based on isoresponses curves was used to find a compromise setting leading to the desired response. In addition, the “desirability” function was used to find the exact optimal setting with a compromise percentage. A value of 0 is awarded when the measured metrics fall below predefined minimum values, suggesting an unacceptable response. Conversely, 100% represents achieving or exceeding all desired performance [58].

### 2.6. Statistical Processing of Data

Statistical analyses were performed using the log-probit program provided by Agricultural Research Centre for International Development - Annual Crops (CIRAD-CA)/ Applied Models for Biopesticides and Insecticide Selectivity (MABIS) [59]. Finney's mathematical procedures were applied to calculate lethal concentration levels (LC_50_ and LC_90_), along with their 95% confidence limits, and to perform the Chi-squared (*χ*^2^) test for goodness of fit. The experimental treatments for the mixture design were planned and analyzed using the DESIGN EXPERT software (Version 13, Stat-Ease, Minneapolis, Minnesota, United States). One-way ANOVA F-test and Tuckey test were used to examine statistically significant differences between mean values, considering a probability of *p* ≤ 0.05 as statistically significant.

## 3. Results

### 3.1. Yield of EOs

**Figure 2:**
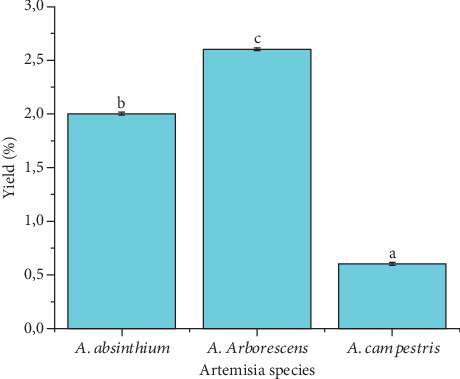
The average yields (%) of the three *Artemisia* species EO. Different letters (a–c) highlighted in each column show a significant difference (*p* < 0.05) according to the Tukey test.

The EO yields varied between 0.6% and 2.6%. *A. arborescens* produced the highest yield (2.6 ± 0.008%), followed by *A. absinthium* (2.0 ± 0.009%), while *A. campestris* yielded the lowest amount (0.6 ± 0.008%).  Figure 2 highlights the statistically significant differences (*p* < 0.05) in EO yields among the three *Artemisia* species.

### 3.2. Chemical Composition of EOs

Table 2 presents the major EO constituents identified in the plants investigated in this study. In the EO of *A. absinthium*, a total of 20 compounds were detected, together accounting for 99.98% of the overall composition. Oxygenated monoterpenes were the predominant class, comprising 57.77 ± 3.20% of the *A. absinthium* EO. The main component was thujone (32.20% ± 2.65%), followed by camphor (19.95% ± 2.64%), chamazulene (19.58 ± 2.33%), and sabinene hydrate (4.27% ± 1.82%). For *A. arborescens*, 35 components were identified, making up 99.99% of the total composition. Oxygenated monoterpenes again dominated, representing 92.04% ± 3.51% of the EO. Thujone was the most abundant compound (52.05% ± 3.84%), followed by camphor (10.71% ± 3.08%), eucalyptol (4.79%% ± 1.53%), and car-3-en-5-one (3.55% ± 1.04%). In the EO of *A. campestris*, 45 compounds were detected, constituting 99.98% of the overall composition. Oxygenated monoterpenes were the major class, accounting for 70.83% ± 4.61%. The principal components included camphor (18.98% ± 2.65%), car-3-en-5-one (11.25% ± 2.33%), thujone (6.63% ± 1.67%), and chrysanthenone (6.24% ± 1.42%).

### 3.3. Larvicidal Activity of EOs Alone on *Culex pipiens*

The larvicidal efficacy of EOs derived from three distinct plant species was evaluated against *C. pipiens*, as detailed in Table 3. The results revealed varying mortality percentages at different concentrations. *A. arborescens* exhibited the highest larvicidal activity, with a calculated LC_50_ of 11.11 *μ*g/mL (5.45 ± 22.62), followed by *A. absinthium* with an LC_50_ of 16.98 *μ*g/mL (6.73 ± 27.39), and *A. campestris* with an LC_50_ of 19.07 *μ*g/mL (13.57 ± 23.38). These findings highlight the differing potencies of the EOs in inducing mortality among *C. pipiens* larvae, with *A. arborescens* demonstrating the most pronounced larvicidal effect.

### 3.4. Optimization of Insecticidal Activity by Mixture Design

#### 3.4.1. Insecticide Formulation Design

The different combinations of the three EOs studied, along with the observed responses in each experiment, are listed in Table 4. The experiments were conducted following randomization, and each response represents the average of three replicates. Before analyzing the mixture design, the results showed that the equiproportional combination of the three oils (66.67% *A. absinthium*–16.67% *A. arborescens*–16.67% *A. campestris*) and the binary combination (50% *A. absinthium*–50% *A. campestris*) exhibited higher mortality than each EO used individually.

#### 3.4.2. Mathematical Model Selection

To identify the model that best captures the observed responses, this study employed linear, quadratic, and special cubic models to fit experimental data and generate regression equations. A thorough analysis of model summary statistics (Table 5) pinpointed the most suitable model for accurate representation.  Table 6 revealed that the linear and special cubic models underperformed compared to the quadratic model in terms of *R*^2^, adjusted *R*^2^, predicted *R*^2^, and *p* values. Therefore, the quadratic model with linear and binary interaction terms was chosen to best explain the effect of the three mixture components on mortality.

#### 3.4.3. Statistical Validation of the Quadratic Model

**Figure 3:**
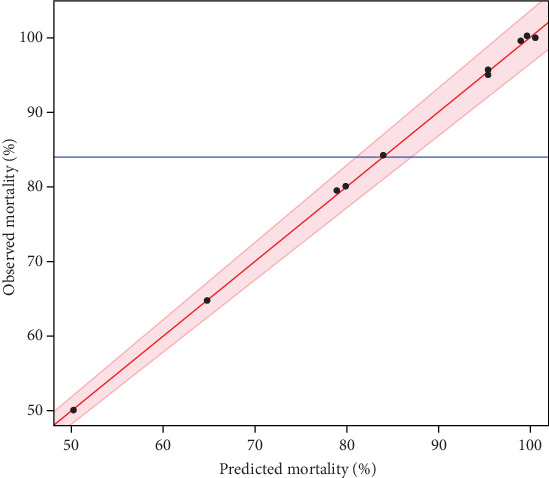
Curves of observed values versus predicted values of the response mortality.

According to the analysis of the variance table (Table 6), the main effect of the regression is significant since the probability of risk significance *p* value is less than 0.05 (< 0.0001⁣^∗^). Furthermore, the models do not show a LOF since the probability of significance of the *p* value risk is greater than 0.05 (0.351). The *R*^2^ adjusted *R*^2^, and predicted R^2^ were very sufficient with 0.997, 0.994, and 0.995, respectively. These values indicate sufficient agreement between the experimental and predicted values of the adapted model. The graph (Figure 3) shows that the curves of observed values versus predicted values are perfectly straight.

#### 3.4.4. Compound Effects and Fitted Model

The effects of all the factors studied were determined using the *t*-student test, with a significance level of 95% reported in Table 7. According to this table, all quadratic model coefficients are statistically significant, with a *p* value of less than 0.05. Therefore, the proposed model had to include all coefficients.

The mathematical model used is presented by the following equation:

(5) $$\begin{array}{c} \text{Y}=79,89\;{\text{X}}_{1}+50,18\;{\text{X}}_{2}+64,81\;{\text{X}}_{3}+136,32\;{\text{X}}_{1}{\text{X}}_{2}+109,13\;{\text{X}}_{1}{\text{X}}_{3}+28,77\;{\text{X}}_{2}{\text{X}}_{3}+ɛ \end{array}$$

#### 3.4.5. Formulation Optimization and Desirability Study

**Figure 4:**
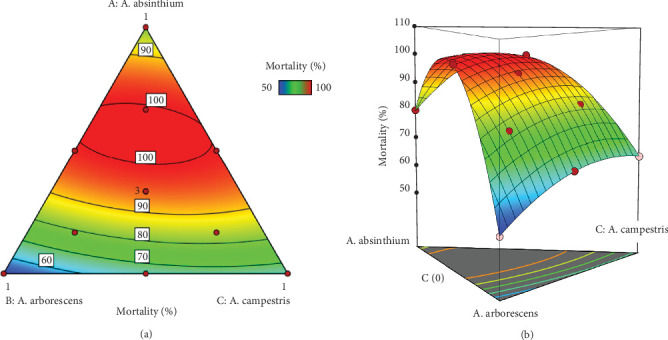
(a) 2D and (b) 3D mixture profiles showing the maximization area of the mortality response as a function of the three components *Artemisia absinthium*, *A*. *arborescens*, and *A*. *campestris* EOs.

**Figure 5:**
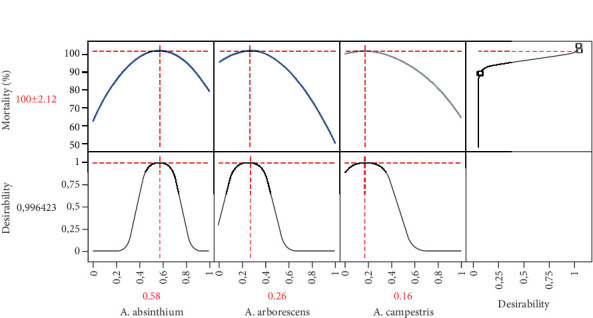
Desirability plot indicating the precise proportions of the three EOs *Artemisia absinthium*, *A*. *arborescens*, and *A. campestris* leading to the maximal mortality.

The aim of this section is to find the optimum formulation of the three EOs that lead to total mortality. As shown in the compromise zone (red areas) in the 2D and 3D contour plots (Figure 4), the optimum zone exists in the center of the triangle, implying that we can achieve a mortality of 100 by ensuring a mixture of all three EOs. In addition, the desirability plot (Figure 5) allows us to determine with precision the proportions of the three oils leading to the optimal value of 100% of mortality. Thus, we have a 99% chance of obtaining this value, which is possible by ensuring a mixture with proportions of 58% *A. absinthium*, 26% *A. arborescens*, and 16% *A. campestris*.

## 4. Discussion

In our investigation, the yield of EO from *A. absinthium* was found to be 2 ± 0.2%, which notably exceeds yields reported in China (0.15 ± 0.01%) [61] and Serbia (0.15 ± 0.03%) [62]. This yield is closer to the 1.5% documented in the Setif region of Algeria [37]. In comparison, the EO yield of *A. campestris* (0.6 ± 0.05%) was lower than that reported in Portugal (1% ± 0.05%) [63] but higher than the yield observed in Serbia (0.180% ± 0.02%) [62]. For *A. arborescens*, the hydrodistilled EO yield was 2.5% ± 0.2%, surpassing yields reported in Libya (0.75 ± 0.1%) [64], Lebanon (1.7% ± 0.1%) [65], and Turkey (1.2 ± 0.1%) [66].

These differences in yield can be attributed to various factors, such as plant species characteristics, the vegetative stage of the plant, its geographical origin [67], and the edaphic conditions of the region [68].

The chemical composition of EOs from different *Artemisia* species has been extensively studied, revealing variations when compared to the current study. A comprehensive analysis of *A. absinthium* from different geographic regions identified 68 compounds, constituting 93.4% of the total oil mass. In Greece, the EO was dominated by caryophyllene oxide (25.3%), *p*-cymene (16.8%), 1,8-cineole (8.9%), and (*Z*)-lanceol acetate (7.3%) [69]. In Iran and China, other primary components were reported, including *β*-thujone (35.1%), *p*-cymene (16.5%), *β*-pinene (7.3%), and 7-ethyl-5,6-dihydro-1,4-dimethylazulene (5.5%) [70], and *β*-myrcene (10.05%), eucalyptol (25.59%), and linalool (11.99%) in China [61]. In Serbia [62], thujone-2,4(10)-diene (15.9%), caryophyllene oxide (14.9%), and *β*-thujone (12.8%) predominated. Our findings align with prior studies emphasizing thujone, camphor, and chamazulene as major components in *A. absinthium* and *A. arborescens* EOs [62, 66, 71]. However, our *A. arborescens* EO lacked chamazulene, a component known for contributing to the oil's dark blue color in *A. absinthium* [61].

The variations in chemical profiles may be influenced by factors such as sexual polymorphism, genetic mechanisms, and external environmental conditions that affect the biosynthetic pathways of the plants [72]. In the EO of *A. campestris*, a study by Abidi et al. [73] identified 50 compounds, with four major ones: *β*-pinene (36.40%), 2-undecanone (14.7%), limonene (10.57%), and benzene (6.3%). The prevalence of *β*-pinene in our findings is consistent with results from Tunisia [74, 75] and Portugal [63] but differs from those reported in Morocco [76] and Algeria [77], where spathulenol (10.19%) and *β*-myrcene (16.47%) were the dominant compounds, respectively.

The discrepancies in the chemical compositions of EOs from *Artemisia* species can largely be attributed to factors such as geographical origin and rainfall, which play a significant role in the biosynthesis of secondary metabolites in plants [78, 79]. These environmental conditions influence the chemical profiles of EOs, leading to differences in the compositions reported across different regions.

Artemisia species, with over 400 species in the Asteraceae family, are well recognized for their medicinal properties, attributed to their diverse phytochemical composition [80]. Our study demonstrates the significant larvicidal activity of EOs from *A. absinthium*, *A. campestris*, and *A. arborescens* against *C. pipiens* larvae, with efficacy increasing over extended exposure times. This finding is noteworthy, as limited literature exists on the larvicidal properties of these EOs against *C. pipiens*, prompting comparisons with results on other *Culicidae* species.

The EO of *A. absinthium* exhibited the highest larvicidal activity, surpassing the efficacy reported in previous studies from India on various mosquito species, including *Anopheles stephensi*, *Anopheles subpictus*, *Aedes aegypti*, *Aedes albopictus*, *Culex quinquefasciatus*, and *Culex tritaeniorhynchus*. Those studies reported LC_50_ values ranging from 41.85 to 62.16 *μ*g/mL [81], whereas our findings demonstrate enhanced efficacy due to the presence of major compounds such as eucalyptol, linalool, and *β*-myrcene, all known for their strong toxicological properties [82–84]. In contrast, *A. campestris* EO displayed the lowest larvicidal activity against *C. pipiens* (LC_50_ = 19.07 *μ*g/mL (13.57–23.38)), though still superior to the activity of methanolic extracts of the same plant against *C. quinquefasciatus* larvae (LC_50_ = 23 ppm (21–24)) as reported previously [85]. Similarly, our results outperform those from Algeria, which recorded an LC_50_ of 45.8 mg/L for *A. campestris* EO against *C. quinquefasciatus* [86].

In the context of EO combinations, our study revealed substantial variations in larvicidal efficacy. Mixtures demonstrated robust insecticidal activity, with several achieving 100% larval mortality. Notably, Experiment 4, which combined *A. absinthium* and *A. arborescens* in equal proportions (0.5 + 0.5), exhibited 99.41% mortality, highlighting a possible synergistic interaction. Similarly, Experiment 5, involving an equal mixture of *A. absinthium* and *A. campestris* (0.5 + 0.5), achieved complete larval eradication. However, Experiment 6, which combined *A. arborescens* and *A. campestris* (0.5 + 0.5), resulted in a lower mortality rate of 64.76%, suggesting potential antagonistic interactions between certain compounds. Experiments 7, 10, 11, and 12, involving varying proportions of all three EOs, exhibited variable mortality rates, reflecting the complex nature of larval sensitivity to these combinations.

These findings align with previous studies validating the effectiveness of aromatic compounds in both individual and combined forms. For instance, Pavela [43] reported synergistic effects of binary combinations of aromatic compounds against *C. quinquefasciatus* larvae, while Hategekimana and Erler [87] explored the repellent activity of plant EOs and their major constituents. Similarly, Boukraa et al. [88] highlighted the insecticidal and repellent potential of synergistic EO combinations. Conversely, Wangrawa et al. [89] documented both synergistic and antagonistic effects of EO blends from *Cymbopogon schoenanthus*, *Lantana camara*, *Lippia chevalieri*, and *Lippia multiflora* against *Anopheles gambiae* larvae and adults.

In line with these studies, our findings suggest that synergistic interactions among EO compounds contribute significantly to enhanced larvicidal activity, as observed in blends containing *A. absinthium*. The consistent efficacy of these mixtures may stem from the presence of potent larvicidal compounds, such as eucalyptol and linalool, in *A. absinthium* oil. Conversely, the reduced activity in certain mixtures, such as Experiment 6, may be attributed to antagonistic interactions among specific compounds. These results underscore the complex interplay of EO constituents and their unpredictable modulation of mixture efficacy. By elucidating the intricate dynamics of these interactions, our study highlights the potential of EO combinations as effective larvicidal agents and provides a basis for optimizing their application in mosquito control programs.

## 5. Conclusions

In this study, we assessed the insecticidal properties of EOs extracted from *Artemisia absinthium*, *Artemisia arborescens*, and *Artemisia campestris* against *Culex pipiens* larvae, both individually and in ternary combinations. The chemical analysis revealed that oxygenated monoterpenes were the dominant class in all three species but with notable differences in their specific compositions. The EO of *A. absinthium* was predominantly composed of thujone (32.20% ± 2.65%), camphor (19.95% ± 2.64%), chamazulene (19.58% ± 2.33%), and sabinene hydrate (4.27% ± 1.82%), with oxygenated monoterpenes accounting for 57.77% ± 3.20% of the total composition. Similarly, *A. arborescens* exhibited a high content of oxygenated monoterpenes (92.04% ± 3.51%), with thujone as the major compound (52.05% ± 3.84%), followed by camphor (10.71 ± 3.08%), eucalyptol (4.79% ± 1.53%), and car-3-en-5-one (3.55% ± 1.04%). *A. campestris* also showed a high proportion of oxygenated monoterpenes (70.83% ± 4.61%), with camphor (18.98% ± 2.65%), car-3-en-5-one (11.25% ± 2.33%), thujone (6.63% ± 1.67%), and chrysanthenone (6.24% ± 1.42%) as the major components. The insecticidal potential of these EOs was further explored using a mixture design approach, where ternary combinations of the oils were tested. The formulation consisting of 58% *A. absinthium*, 26% *A. arborescens*, and 16% *A. campestris* demonstrated the highest larvicidal efficacy, resulting in the complete eradication of *C. pipiens* larvae. This increased effectiveness can be attributed to the synergistic interactions between the various monoterpene and sesquiterpene compounds in the oils, along with the specific proportions used in the mixture.

These results highlight the promising potential of indigenous *Artemisia* species for mosquito control and emphasize the importance of refining EO formulations to enhance their larvicidal activity. This study contributes valuable insights for future research focused on the development of sustainable, plant-based insecticides.

## Figures and Tables

**Table 1:** Primary data from the study evaluating the larvicidal activity of essential oils on tested plant species.

**Species**	**Harvest date**	**Site**	**GPS coordinates**	**Altitude (m)**
*A. absinthium*	April 2022	Moulay Yacoub (Moulay Yacoub Province)	34°17⁣′05.3⁣^″^ N 5°00⁣′53.9⁣^″^ W	196
*A. campestris*	May 2022	Lmarija (Guercif Province)	33°58⁣′56.0⁣^″^ N 3°17⁣′31.9⁣^″^ W	816
*A. arborescens*	June 2022	Talzemt (Boulemane Province)	33°36⁣′47⁣^″^ N 4°11⁣′30⁣^″^ W	1921

*Note:* Details regarding the plant collection process, including specific procedures for harvesting, transport, and preparation of the plant material, are described in the Material and Methods section.

**Table 2:** Chemical composition of EOs obtained from *Artemisia* species obtained by GC-MS.

**No.**	**Compounds**	**Formula**	**RI** ^**a**^	**RI Lit** ^**b**^	**% relative peak area**		
					***A. absinthium***	***A. arborescens***	***A. campestris***
1	Sabinene	C_10_H_16_	897	973	0.82 ± 0.03	2.02 ± 1.02	—
2	Camphene	C_10_H_16_	943	946	—	1.76 ± 0.05	2.46 ± 1.01
3	*β*-Myrcene	C_10_H_16_	958	974	1.66 ± 0.41	—	—
4	*γ*-Terpinene	C_10_H_16_	998	1022	0.57 ± 0.32	0.47 ± 0.15	—
5	Sabina ketone	C_9_H_14_O	1001	1132	—	0.83 ± 0.19	—
6	Pseudolimonene	C_10_H_16_	1013	1010	—	0.36 ± 0.04	—
7	Hemellitol	C_9_H_12_	1020	1018	—	—	1.68 ± 0.08
8	Sabinene hydrate	C_10_H_18_O	1041	1039	4.27 ± 1.82	—	—
9	*O*-Cymene	C_10_H_14_	1042	1066	—	1.39 ± 0.22	1.74 ± 0.54
10	Eucalyptol	C_10_H_18_O	1059	1057	—	4.79 ± 1.53	3.45 ± 1.65
11	Thujone	C_10_H_16_O	1062	1059	32.20 ± 2.65	52.05 ± 3.84	6.63 ± 1.67
12	*Cis*-Thujanol	C_10_H_18_O	1085	1078	—	1.33 ± 0.53	—
13	Cyclohexene, 2-ethenyl-1,3,3-trimethyl-	C_11_H_18_	1105	1048	0.41 ± 0.02	—	—
14	*P*-Menth-2-en-1-ol	C_10_H_18_O	1109	1115	—	—	1.74 ± 0.05
15	Ocimenone	C_10_H_14_O	1112	1225	—	0.31 ± 0.01	1.57 ± 0.08
16	Pinocarvone	C_10_H_14_O	1114	1124	—	0.50 ± 0.02	—
17	Neoallocimene	C_10_H_16_	1118	1117	—	—	0.40 ± 0.01
18	Car-3-en-5-one	C_10_H_14_O	1119	1114	—	3.55 ± 1.04	11.25 ± 2.33
19	Filifolone	C_10_H_14_O	1119	1117	—	—	4.56 ± 1.36
20	Chrysanthenone	C_10_H_14_O	1119	1123	—	—	6.24 ± 1.42
21	Verbenone	C_10_H_14_O	1119	1177	—	0.72 ± 0.88	—
22	Camphor	C_10_H_16_O	1121	1117	19.95 ± 2.64	10.71 ± 3.08	18.98 ± 2.65
23	Myrtenal	C_10_H_14_O	1136	1136	—	0.83 ± 0.03	—
24	Chrysanthenol	C_10_H_16_O	1136	1134	—	—	2.46 ± 1.21
25	Terpinen-4-ol	C_10_H_18_O	1137	1164	1.35 ± 0.18	1.84 ± 0.51	1.10 ± 0.09
26	Borneol	C_10_H_18_O	1138	1136	—	2.94 ± 0.86	3.56 ± 1.22
27	2,5-Dimethylbicyclo [3.3.0] oct-6-en-8-one	C_10_H_14_O	1150	1148	—	0.28 ± 0.01	—
28	Piperitone	C_10_H_16_O	1158	1221	—	0.43 ± 0.02	2.63 ± 1.05
29	Carvotanacetone	C_10_H_16_O	1158	1213	—	0.37 ± 0.01	0.71 ± 0.03
30	Piperitol	C_10_H_18_O	1175	1176	—	—	2.58 ± 1.00
31	(+) -2-(1-Hdroxyethyl) apopinene	C_11_H_18_O	1210	1410	—	—	1.54 ± 0.08
32	4-Hydroxy-*β*-thujone	C_10_H_16_O_2_	1213	1116	—	2.28 ± 1.02	—
33	*Trans*-Sabinyl acetate	C_10_H_18_O_2_	1224	1265	—	2.97 ± 1.01	—
34	Butylphenyl	C_10_H_14_O	1228	1500	—	—	0.38 ± 0.01
35	Cuminaldehyde	C_10_H_12_O	1230	1226	—	0.80 ± 0.02	—
37	1,4-p-Menthadien-7-ol	C_10_H_16_O	1240	1240	—	0.27 ± 0.01	—
38	Chrysanthemic acid	C_10_H_16_O_2_	1256	1283	—	0.64 ± 0.3	—
39	Cyclopropane carboxylic acid, 2,2-dimethyl-3-	C_10_H_16_O_2_	1256	1283	—	—	0.40 ± 0.01
40	Thymol	C_10_H_14_O	1262	1282	—	0.48 ± 0.02	—
41	Chrysanthenyl acetate	C_12_H_18_O	1276	1274	—	—	3.84 ± 1.21
42	Fenchyl acetate	C_12_H_20_O_2_	1277	1253	—	—	1.16 ± 0.09
43	Cuminol	C_10_H_14_O	1284	1275	—	0.53 ± 0.03	—
44	Filifolide A	C_10_H_14_O_2_	1293	1318	—	0.67 ± 0.02	2.14 ± 1.09
45	Rosefuran	C_10_H_14_O	1341	1115	—	—	0.45 ± 0.02
46	Copaen-4-∝-ol	C_15_H_24_O	1367	1576	—	—	0.60 ± 0.02
47	Isobornyl acrylate	C_13_H_20_O_2_	1390	1374	—	—	1.50 ± 0.08
48	Chamazulene	C_14_H_16_	1455	1444	19.58 ± 2.33	—	—
49	Dihydrochamazulene	C_15_H_20_	1455	1651	1.34 ± 0.09	—	—
50	Alloaromadendrene oxide	C_15_H_24_O	1462	1702	1.30 ± 0.08	—	—
51	1-Acetoxy-p-menth-3-one	C_13_H_20_O_3_	1488	1471	—	0.35 ± 0.02	1.44 ± 0.09
52	Caryophyllene	C_15_H_24_	1494	1467	0.79 ± 0.04	—	—
53	Caryophyllene oxide	C_15_H_24_O	1507	1572	—	0.24 ± 0.01	—
54	Germacrene D	C_15_H_24_	1515	1510	3.88 ± 1.04	0.53 ± 0.03	0.54 ± 0.02
55	Elemol	C_15_H_26_O	1522	1541	0.82 ± 0.06	—	—
56	Ledol	C_15_H_26_O	1530	1565	—	—	4.23 ± 1.42
57	Palustrol	C_15_H_26_O	1530	1548	—	—	0.65 ± 0.07
58	Spathulenol	C_15_H_24_O	1536	1575	—	—	2.19 ± 1.06
59	2,4-di-tert-butylphenol	C_14_H_22_O	1555	1502	1.11 ± 0.09	—	—
60	Rosifoliol	C_15_H_26_O	1598	1603	4.15 ± 1.82	—	—
61	Xanthoxylin	C_10_H_12_O_4_	1628	1625	—	2.75 ± 1.01	—
62	*Cis*-Carvyl tiglate	C_12_H_18_O_2_	1629	1629	—	—	3.07 ± 1.67
63	1H-Benzocyclohepten-7-ol, 2,3,4,4a,5,6,7,8-octahy	C_15_H_26_O	1651	1618	—	—	0.77 ± 0.02
64	Globulol	C_15_H_26_O	1681	1604	—	—	0.61 ± 0.08
65	Arborescin	C_15_H_20_O_3_	1695	1742	1.64 ± 0.09	—	—
**66**	Geranyl-*α*-terpinene	C_15_H_32_	1962	1955	3.33 ± 1.44	—	—
**67**	3-Methyl-2-butenoic acid, tridec-2-ynyl ester	C_18_H_30_O_2_	1980	2035	—	—	0.74 ± 0.35
	Monoterpene hydrocarbons				3.05 ± 1.08	6.00 ± 3.15	4.60 ± 1.68
	Oxygenated monoterpenes				57.77 ± 3.20	92.04 ± 3.51	70.83 ± 4.61
	Sesquiterpene hydrocarbons				9.34 ± 2.71	0.53 ± 0.04	0.54 ± 0.02
	Oxygenated Sesquiterpenes				7.91 ± 2.97	0.24 ± 0.01	9.66 ± 1.24
	Others				21.1 ± 2.89	1.18 ± 0.09	14.97 ± 3.24
	Total identified %				99.17 ± 0.61	99.99 ± 0.01	99.99 ± 0.01

*Note:* Data are expressed as the mean standard deviation of three replicates. Retention indices (RI^a^) are experimentally calculated retention indices for a homologous series of C8-C28 alkanes. Literature-based retention indices (RI Lit^b^) [60]; −: absence; data is expressed as mean ± standard deviation of triplicates.

**Table 3:** Lethal concentrations of the three plants' EO (LC_50_ and LC_90_).

**Plant species**	**LC **_**50**_ ** (*μ*g/mL) (Ll-Ul)**⁣^∗^	**LC **_**90**_ ** (*μ*g/mL) (Ll-Ul)**⁣^∗^	**Equation of the regression line**	**Calculated chi2**
*A. absinthium*	16.98 (6.73 ± 27.39)	60.79 (37.43 ± 164.99)	*Y* = −2.84751 + 2.31480∗*X*	15.204
*A. arborescens*	11.11 (5.45 ± 22.62)	23.65 (14.18 ± 39.45)	*Y* = −4.08416 + 3.90538∗*X*	10.297
*A. campestris*	19.07 (13.57 ± 23.38)	35.63 (28.37 ± 60.46)	*Y* = −6.04918 + 4.72392∗*X*	13.871

*Note:* LC_50_ = lethal concentration capable of killing 50% of exposed larvae; LC_90_ = lethal concentration capable of killing 90% of the exposed larvae.

**Table 4:** Different combinations produced by the augmented simplex-centroid design and observed response.

**Run** ^**a**^	**Mixture**			**Observed response values** ^**b**^
	***A. absinthium***	***A. arborescens***	***A. campestris***	**Mortality (%)**
1	1	0	0	80.00 ± 3.21
2	0	1	0	50.00 ± 1.45
3	0	0	1	64.70 ± 3.85
4	0.5	0.5	0	99.41 ± 1.03
5	0.5	0	0.5	100.00 ± 0.00
6	0	0.5	0.5	64.76 ± 4.23
7	0.33	0.33	0.33	95.60 ± 2.56
8	0.33	0.33	0.33	95.12 ± 2.84
9	0.33	0.33	0.33	95.00 ± 2.65
10	0.67	0.17	0.17	100.00 ± 0.00
11	0.17	0.67	0.17	79.35 ± 3.47
12	0.17	0.17	0.67	84.18 ± 2.69

^a^Runs are realized after randomization.

^b^The observed value of three replicates is listed with the standard deviation.

**Table 5:** Model summary statistics.

**Source**	**Sequential ** **p** ** value**	**Lack of fit ** **p** ** value**	**R** ^2^	**Adjusted ** **R** ^2^	**Predicted ** **R** ^2^	**Remarks**
Linear	0.1126	0.0004	0.385	0.2478	−1.0281	
**Quadratic**	**< 0.0001**	**0.351**	**0.9997**	**0.9994**	**0.9956**	**Suggested**
Cubic	0.2187	0.3779	0.9998	0.9991	0.994	

*Note:* Bold entries highlight the suggested model based on the statistical criteria, including the highest *R*^2^, adjusted *R*^2^, and predicted *R*^2^ values, along with a nonsignificant lack of fit *p* value.

**Table 6:** Analysis of variance for the adjusted model.

**Source**	**Degrees of freedom**	**Sum of squares**	**Mean square**	**F** ** value**	**p** ** value**
Model	5	3063.9666	612.793	3544.849	< 0.0001⁣^∗^
Error	6	1.0372	0.173		
Total	11	3065.0038			
Lack of fit	4	0.8356121	0.208903	2.0725	0.351
Pure error	2	0.2016000	0.100800		
*R* ^2^	0.9997				
*R*_adj_^2^	0.9994				
*R*_pred_	0.9956				

⁣^∗^Statistically significant at *p* < 0.05.

**Table 7:** Estimated regression coefficients for the fitted model.

**Terms**	**Coefficient**	**Estimate**	**Standard error**	**t** ** -student**	**p** ** value**
*A. absinthium*	*α*1	79.890404	0.398987	200.23	< 0.0001⁣^∗^
*A. arborescens*	*α*2	50.189495	0.398987	125.79	< 0.0001⁣^∗^
*A. campestris*	*α*3	64.816768	0.398987	162.45	< 0.0001⁣^∗^
*A. absinthium *⁣^∗^* A. arborescens*	*α*12	136.32424	1.78465	76.39	< 0.0001⁣^∗^
*A. absinthium *⁣^∗^* A. campestris*	*α*13	109.13879	1.78465	61.15	< 0.0001⁣^∗^
*A. arborescens *⁣^∗^* A. campestris*	*α*23	28.77697	1.78465	16.12	< 0.0001⁣^∗^

⁣^∗^Statistically significant at *p* < 0.05.

## Data Availability

All related data are contained within the manuscript.
